# Research advances in extracellular vesicles for diagnosis and treatment of genitourinary cancers

**DOI:** 10.3389/fcell.2026.1793573

**Published:** 2026-03-12

**Authors:** Minghong Zhao, Wenlong Huang, Changwei Yang, Linke Lu, Linli Chen, Jun Wang, Hao Yang, Qiuyan Guo, Tao Qin, Defa Huang

**Affiliations:** 1 Laboratory Medicine, Guizhou Aerospace Hospital, Zunyi, China; 2 Department of General Medicine, The First People’s Hospital of Zunyi (Third Affiliated Hospital of Zunyi Medical University), Zunyi, China; 3 Department of Nuclear Medicine, The Affiliated Hospital of Zunyi Medical University, Zunyi, China; 4 Sate Key Laboratory for Quality Ensurance and Sustainable Use of Dao-di Herbs, Artemisinin Research Center, and Institute of Chinese Materia Medica, Academy of Chinese Medical Sciences, Beijing, China; 5 Laboratory Medicine, The First Affiliated Hospital of Gannan Medical University, Ganzhou, China

**Keywords:** biomarkers, diagnosis, extracellular vesicles, treatment, urogenital cancers

## Abstract

Extracellular vesicles (EVs) have emerged as vital mediators of intercellular communication, playing crucial roles in the initiation, progression, and metastasis of urogenital cancers. Due to their ability to carry diverse biological molecules and their excellent biocompatibility, EVs have garnered significant attention as novel tools for the diagnosis and treatment of malignancies such as prostate, bladder, kidney, and testicular cancers. This review summarizes recent progress in understanding the biological functions of EVs in various urogenital tumors, integrates findings from both fundamental studies and clinical trials, and discusses ongoing obstacles and future prospects in the field. By providing insights into the diagnostic and therapeutic applications of EVs, this article aims to support the development of precision medicine strategies for urogenital cancer patients.

## 1 Introduction

The genitourinary system encompasses organs such as the prostate, bladder, kidney, and testis, which are vulnerable to malignant transformation leading to cancers with significant morbidity and mortality worldwide (Zhang et al., 2025; Tian et al., 2023). Prostate cancer (PCa) is among the most prevalent malignancies in men globally, while bladder and kidney cancers rank highly in incidence and mortality within urinary tract malignancies (Jain et al., 2023; Kadeerhan et al., 2025). Testicular cancer, though less common, predominantly affects younger males and presents unique clinical challenges (Li et al., 2021). Epidemiological analyses reveal increasing incidence trends for these cancers, with notable geographic, racial, and socioeconomic disparities influencing disease burden and outcomes (Tian et al., 2023; Ghazwani et al., 2024). Despite advances in imaging and histopathology, early diagnosis and precise risk stratification remain challenging due to tumor heterogeneity and limitations of current diagnostic modalities (Bertuccio et al., 2021; Katims et al., 2020). Traditional diagnostic approaches for genitourinary cancers often rely on invasive procedures such as tissue biopsies and cystoscopies, which are associated with patient discomfort, risk of complications, and considerable healthcare costs (Brisotto et al., 2021; Yang et al., 2025). For example, serum prostate-specific antigen (PSA) testing, while widely used for PCa screening, suffers from low specificity leading to overdiagnosis and unnecessary biopsies (El Sharkawi et al., 2023; Ballesteros Ruiz et al., 2022). Similarly, urine cytology for bladder cancer exhibits high specificity but poor sensitivity, particularly for low-grade tumors (Yang et al., 2025). These limitations underscore the urgent need for novel, minimally invasive biomarkers that can enhance early detection, monitor disease progression, and guide therapeutic decisions (Oyaert et al., 2025; Urabe et al., 2021).

EVs are nanoscale, membrane-enclosed particles secreted by virtually all cell types into various body fluids, including blood, urine, and saliva. These vesicles encapsulate a diverse array of biomolecules such as proteins, nucleic acids (including mRNAs, microRNAs, long non-coding RNAs, and piRNAs), lipids, and metabolites, thereby serving as critical mediators of intercellular communication (Oliveira et al., 2020; Chen and Ding, 2025). Their biogenesis involves complex cellular processes, resulting in heterogeneous populations classified based on size and origin, including exosomes (30–150 nm), microvesicles, and apoptotic bodies (Papakonstantinou et al., 2024). The lipid composition of EVs not only provides structural integrity but also influences their cargo sorting, cellular uptake, and functional effects on recipient cells (Fyfe et al., 2023; Skotland et al., 2020). Due to their stability in body fluids and ability to reflect the molecular signature of their parental cells, EVs have emerged as promising candidates for non-invasive biomarker discovery and therapeutic delivery systems (Shekari et al., 2021; Jurgielewicz et al., 2021). Urine is an especially attractive biofluid for biomarker discovery in genitourinary cancers due to its direct contact with the urinary tract and ease of collection (Jiang S. et al., 2022; De Matteis et al., 2021). Within urine extracellular vesicles (uEVs) represent a rich source of tumor-derived molecular information, including proteins, various types of RNAs, and metabolites, encapsulated in a protected environment that preserves their stability (Lange et al., 2025; Noriega Landa et al., 2024). Recent proteomic and transcriptomic studies have identified numerous candidate biomarkers in uEVs that differentiate cancer patients from healthy controls and correlate with tumor stage and grade (Tomiyama et al., 2021; Fan et al., 2025). For instance, specific mRNAs, miRNAs, long non-coding RNAs (lncRNAs), and piRNAs in urinary EVs have shown promising diagnostic and prognostic potential for prostate and bladder cancers (Brokāne et al., 2023; Peng et al., 2021; Georgantzoglou et al., 2021). Moreover, glycomic and lipidomic profiling of uEVs have revealed distinct cancer-associated signatures that may further refine biomarker panels (Li Y. et al., 2025; Jadamba et al., 2024). Given the substantial progress in EVs research and the pressing clinical challenges in managing genitourinary cancers, there is a growing emphasis on systematically evaluating the diagnostic and therapeutic applications of EVs in this field.

This review discusses the biological characteristics of EVs and their mechanism of action in tumors. Summarizes the latest advancements in the diagnosis and treatment of urogenital system cancers (including prostate cancer, bladder cancer, kidney cancer, and testicular cancer) using EVs. Furthermore, we also discussed the current challenges faced in this field as well as its future development prospects. This review aims to provide ideas for EVs in the research of urogenital system cancers.

## 2 Biological characteristics of extracellular vesicles and their mechanisms of action in tumors

### 2.1 Classification and biogenesis mechanisms of extracellular vesicles

**FIGURE 1:**
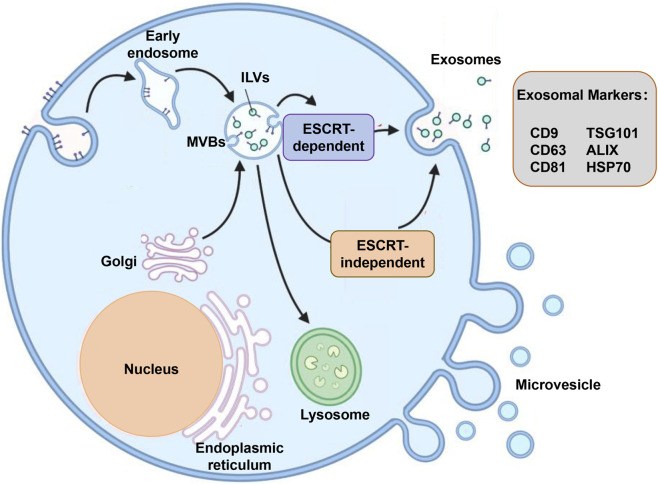
Extracellular vesicles biogenesis. Exosomes are produced by the multivesicular bodies (MVBs). First, the endocytic vesicles on the plasma membrane invaginate to form early endosomes. Then, early endosomes mature into late endosomes and accumulate intraluminal vesicles (ILVs) in their lumen through inward budding of the endocytic membrane during this process. Because of their morphological features, endosomes containing ILVs are generally referred to as MVBs. During formation of MVBs, some bioactive molecules (e.g., proteins, mRNA, miRNA, lncRNA, and circRNA) are packaged into ILVs through special sorting pathways including: 1. the endosomal sorting complex required for transport (ESCRT)-dependent pathway; 2. the ESCRT-independent pathway. Finally, the fusion of MVBs with the plasma membrane results in the release of ILVs into the extracellular space as exosomes.

EVs are a heterogeneous group of membrane-bound particles secreted by virtually all cell types, playing critical roles in intercellular communication by transferring bioactive molecules such as proteins, lipids, and nucleic acids. The primary categories of EVs include exosomes, microvesicles (also referred to as microparticles), and apoptotic bodies, each distinguished by their biogenesis pathways, size, and molecular composition (Figure 1). Exosomes are small vesicles, typically ranging from 30 to 150 nm in diameter, generated intracellularly within multivesicular bodies (MVBs) (Kalluri and LeBleu, 2020). These MVBs fuse with the plasma membrane to release exosomes into the extracellular space. This endosomal origin imparts specific membrane proteins and lipids to exosomes, which can be used as markers to differentiate them from other EV subtypes. Microvesicles, in contrast, are generally larger and originate directly from the outward budding or shedding of the plasma membrane, resulting in vesicles that can vary widely in size, often larger than exosomes (Kalluri and LeBleu, 2020). Apoptotic bodies are formed during programmed cell death and are typically larger than both exosomes and microvesicles, containing cellular fragments including organelles and nuclear material (Kalluri and LeBleu, 2020). The membrane structure of EVs is crucial, as it protects the internal cargo from enzymatic degradation in the extracellular environment, thereby ensuring the stability and fidelity of the transmitted molecular signals. Recent proteomic analyses have revealed that the conventional classification of EVs based solely on size and biogenesis is an oversimplification; both exosomes and microvesicles can exhibit overlapping size ranges and share certain protein markers, reflecting a complex heterogeneity influenced by cellular origin and physiological context. Furthermore, the biogenesis of EVs involves intricate cellular machinery, including the endosomal sorting complexes required for transport (ESCRT) system for exosome formation and cytoskeletal remodeling for microvesicle shedding. Emerging studies also highlight specialized EV subsets, such as mitochondrial extracellular vesicles, which carry mitochondrial components and have distinct functional roles. The selective incorporation of cargo into EVs is tightly regulated, involving specific sorting mechanisms that determine their biological activity and potential as diagnostic biomarkers or therapeutic vehicles. Understanding the detailed biogenesis pathways and molecular characteristics of EV subtypes is essential for exploiting their clinical applications, particularly in the diagnosis and treatment of diseases such as urogenital cancers, where EVs can serve as non-invasive biomarkers or targeted delivery platforms. In summary, EVs encompass a diverse spectrum of vesicles-exosomes, microvesicles, and apoptotic bodies-each with unique biogenetic origins and functional properties, and their membrane-encapsulated cargo ensures stable intercellular communication critical for physiological and pathological processes (Zhang et al., 2020; Liu et al., 2021; Jin et al., 2024; Marostica et al., 2020).

### 2.2 EVs distribution in body fluids and their advantages as liquid biopsy tools

EVs are ubiquitously present in a wide variety of body fluids, including blood, urine, semen, saliva, cerebrospinal fluid, milk, tears, and others, making them readily accessible for clinical sampling and analysis. This broad distribution across easily obtainable fluids is a key advantage for their use as liquid biopsy tools, as it enables non-invasive or minimally invasive collection methods that are more acceptable to patients and suitable for repeated sampling over time. For example, EVs in blood and urine have been extensively studied as sources of biomarkers for cancer and other diseases, while EVs in saliva and tears have shown promise for oral diseases and ocular conditions, respectively (Kano et al., 2021; Wu et al., 2023). The ability to isolate EVs from these diverse fluids facilitates real-time monitoring of disease progression and treatment response without the need for invasive tissue biopsies.

The structural stability of EVs, conferred by their lipid bilayer membrane, protects their molecular cargo-including proteins, nucleic acids (such as miRNAs, piRNAs, mRNAs, DNA), lipids, and metabolites-from enzymatic degradation in the extracellular environment. This stability ensures that EVs maintain the integrity of their cargo during circulation and storage, making them reliable carriers of disease-specific molecular information (Muraoka et al., 2022; Lu et al., 2025). Moreover, EVs carry surface proteins that are reflective of their cell of origin, such as tetraspanins CD9, CD63, and CD81, which facilitate their identification and isolation using affinity-based methods (Giovanazzi et al., 2023; Torsello et al., 2024). The presence of these specific markers enables selective enrichment of EV subpopulations, enhancing the sensitivity and specificity of biomarker detection. Another significant advantage of EVs as liquid biopsy tools is their capacity to reflect the physiological or pathological state of their parental cells. The molecular composition of EVs varies depending on the tissue source and disease condition, allowing for the identification of disease-associated EV signatures. For instance, EVs derived from cancer cells carry oncogenic proteins and nucleic acids that can serve as early diagnostic and prognostic biomarkers (Dabral et al., 2024; Yu et al., 2021). In neurodegenerative diseases, cerebrospinal fluid-derived EVs contain proteins that aid in differential diagnosis and monitoring (Hirschberg et al., 2023). Similarly, EVs in urine have been shown to harbor biomarkers with high diagnostic potential for Alzheimer’s disease and prostate cancer (Cai et al., 2024; Allelein et al., 2022). The heterogeneity of EV surface proteins and cargo also enables the discrimination of EV subpopulations, which may be critical for understanding disease mechanisms and improving diagnostic accuracy (Lopez Baltazar et al., 2024). Furthermore, the non-invasive nature of EV sampling from body fluids allows for repeated measurements, facilitating longitudinal monitoring of disease dynamics and treatment efficacy. This is particularly valuable in oncology, where tumor heterogeneity and evolution necessitate continuous assessment (Yu et al., 2021; Noor et al., 2023). The ability to capture EVs from fluids such as urine and saliva, which are easy to collect and pose minimal discomfort, further enhances their clinical utility (Wu et al., 2023; Hu et al., 2022). Advanced isolation and detection technologies, including automated magnetic particle processing and nanostructured affinity membranes, have improved the throughput, purity, and sensitivity of EV analysis, making large-scale clinical applications feasible (Muraoka et al., 2022; Torsello et al., 2024).

In summary, the widespread distribution of EVs in multiple body fluids combined with their stable structure and disease-specific molecular cargo renders them ideal candidates for liquid biopsy applications. Their accessibility through non-invasive sampling, protection of biomolecules, and the ability to reflect the status of their cells of origin collectively provide significant advantages over traditional biopsy methods. These features position EVs as powerful tools for early diagnosis, prognosis, and therapeutic monitoring across a broad spectrum of diseases, including cancers of the urogenital system. Continued advancements in EV isolation, characterization, and molecular profiling technologies will further enhance their clinical applicability and reliability as liquid biopsy biomarkers (Kano et al., 2021; Muraoka et al., 2022; Dabral et al., 2024). Table 1 summarizes the EVs markers for diagnosing different urinary cancer.

**TABLE 1:** EVs markers in different urinary cancers.

Cancer type	EV biomarkers	Separation method	Sensitivity	Specificity	AUC	References
Prostate cancer	MiR-141 and miR-221	Ultracentrifugation	85%	83%	-	52
	PSA	Ultracentrifugation	-	-	-	54
	miR-375-3p and miR-141	Ultrafiltration	-	-	-	55
Bladder cancer	miR-516A-5P	Ultracentrifugation	72.9	89.9	0.79	67
	Alpha-2-macroglobulin	Ultracentrifugation	93.3	73.2	0.809	68
	lncRNAs (MALAT1, SCARNA10, LINC00963 and LINC01578)	Ultracentrifugation	-	-	0.917	69
Renal cell carcinoma	miR-135b-5p, miR-200c-3p and miR-203a-3p	Reagent kit method	-	-	0.785	95
	miR-320d	Reagent kit method	-	-	-	96
	CD147, CA9, and CD70	Ultracentrifugation	-	-	-	97
	ncRNA AGAP2-AS1	Reagent kit method	-	-	-	102
Testicular cancer	miR-331-3p	Ultracentrifugation	-	-	-	115

## 3 Application of extracellular vesicles in the diagnosis and treatment of prostate cancer

### 3.1 EVs as biomarkers for prostate cancer

EVs, nanosized lipid bilayer-enclosed particles secreted by cells, have emerged as promising sources of biomarkers for PCa diagnosis due to their ability to carry molecular cargo reflective of their cells of origin. Multiple studies have identified that EVs isolated from biological fluids such as blood and urine of PCa patients harbor elevated levels of specific miRNAs and proteins that are associated with tumor presence and progression. For instance, miR-21 and miR-141, two miRNAs frequently implicated in oncogenic processes, have been consistently found at significantly higher concentrations in EVs derived from PCa patients compared to controls (Li et al., 2023; Han et al., 2022). Similarly, prostate cancer antigen 3 (PCA3) and prostate-specific antigen (PSA), proteins traditionally used in PCa screening, are also enriched in EVs from patient samples, suggesting that EV-associated forms of these markers may enhance diagnostic accuracy (Lange et al., 2025; Pang et al., 2020).

The advantages of using EV-based biomarkers lie in their stability in circulation and their capacity to reflect the molecular heterogeneity of tumors more comprehensively than tissue biopsies. Urinary EVs (U-EVs) have been particularly highlighted for their non-invasive accessibility and their direct contact with the prostate, making them an excellent source for detecting prostate-derived biomarkers. Proteomic analyses of U-EVs from patients with varying Gleason scores have revealed distinct protein signatures linked to tumor aggressiveness and disease pathways such as inflammatory responses, cell adhesion, and apoptosis, which are not observed in healthy controls (Lange et al., 2025). Moreover, the miRNA content of urinary EVs, including miR-375-3p and miR-141, has shown potential in distinguishing PCa from benign prostatic hyperplasia (BPH) and in monitoring treatment responses (Bajo-Santos et al., 2023). Combining EV biomarkers with traditional diagnostic methods has demonstrated improved sensitivity and specificity for early detection of clinically significant prostate cancer. For example, the integration of EV-derived markers with multiparametric MRI (mpMRI) and PSA density measurements has yielded higher predictive accuracy for identifying aggressive tumors, thereby reducing unnecessary biopsies (Britton et al., 2025; Hamed et al., 2024). Additionally, panels of EV-associated RNAs and proteins have outperformed individual markers, with pooled sensitivity and specificity reaching above 80% in meta-analyses (Li et al., 2023). This synergistic approach leverages the molecular richness of EV cargo and the anatomical insights from imaging to refine patient stratification.

Technological advancements in EV isolation and characterization, such as chemical affinity capture and immunomagnetic enrichment targeting prostate-specific membrane antigen (PSMA) or other prostate-derived surface markers, have enhanced the purity and yield of prostate-specific EVs from complex biofluids (Allelein et al., 2022; Zeng et al., 2025). These improvements facilitate more reliable downstream analyses, including proteomics and nucleic acid profiling, which are critical for validating candidate biomarkers and translating them into clinical assays.

Despite the promising progress, challenges remain, including the heterogeneity of EV populations, variability in isolation protocols, and the need for standardized methods to ensure reproducibility and comparability across studies (Chan et al., 2025; Ramirez-Garrastacho et al., 2022). Furthermore, the dynamic nature of EV cargo in response to tumor progression and treatment necessitates longitudinal studies to establish robust biomarkers for early diagnosis, prognosis, and therapeutic monitoring. In summary, the research landscape indicates that EVs carrying specific miRNAs such as miR-21 and miR-141, and proteins including PCA3 and PSA, are significantly elevated in the blood and urine of prostate cancer patients. When combined with conventional diagnostic tools, EV-based biomarkers enhance the sensitivity and specificity of early prostate cancer detection. Continued refinement of EV isolation techniques and comprehensive molecular characterization will be pivotal in advancing EV biomarkers toward routine clinical application for prostate cancer diagnosis and management.

### 3.2 The potential of EVs in targeted therapy for prostate cancer

EVs have emerged as promising nanocarriers for targeted therapy in PCa, offering advantages such as biocompatibility, low immunogenicity, and enhanced permeability. One major therapeutic strategy involves utilizing EVs as drug delivery vehicles to achieve targeted transport of chemotherapeutic agents like docetaxel, thereby improving efficacy while minimizing systemic toxicity. For instance, nanovesicles derived from induced pluripotent stem cell-derived mesenchymal stem cells (iPSC-MSCs) have demonstrated selective uptake by prostate cancer cells *in vitro* and *in vivo*, outperforming conventional liposomes in tumor accumulation. These nanovesicles efficiently encapsulate docetaxel, showing enhanced cytotoxicity against docetaxel-resistant PCa cells and reduced toxicity to non-tumor cells. In mouse models of subcutaneous and bone metastatic PCa, docetaxel-loaded nanovesicles significantly suppressed tumor growth and mitigated hematologic toxicity compared to free docetaxel, underscoring their potential to improve therapeutic outcomes in metastatic prostate cancer (Zhao et al., 2021). Similarly, EVs engineered to express human Fcγ receptor I (hCD64) and conjugated with anti-prostate-specific membrane antigen (PSMA) antibodies have been shown to specifically target PSMA-expressing prostate cancer cells and xenografts, highlighting the feasibility of EV-based targeted delivery systems that exploit tumor-specific antigens (Kim et al., 2024). Beyond drug delivery, EVs also mediate gene therapy approaches by transporting nucleic acids such as siRNAs and miRNAs to modulate tumor-associated gene expression. For example, miRNA Let-7b, a known tumor suppressor, is downregulated in PCa cells but enriched in their EVs, which can transfer this miRNA to tumor-associated macrophages, potentially influencing the tumor microenvironment and immune response (Costanzi et al., 2020). Moreover, miR-26a has been identified to regulate EV secretion in PCa cells by targeting genes involved in vesicle biogenesis, and its modulation affects tumor progression, suggesting that EV-mediated miRNA delivery could be harnessed to alter tumor behavior (Urabe et al., 2020). The delivery of miRNAs via EVs also holds promise in overcoming therapy resistance; for instance, miR-200c-3p downregulation in radioresistant PCa cells and their EVs contributes to enhanced DNA repair, and restoring its expression sensitizes cells to radiation, indicating EV-mediated miRNA therapy as a strategy to counteract resistance (Labbé et al., 2024). Furthermore, mesenchymal stem cell-derived EVs have been explored as vehicles to deliver therapeutic agents and modulate the tumor microenvironment, showing potential to induce anti-tumor immune responses and improve safety profiles compared to conventional therapies (Lavi Arab et al., 2024). The unique ability of EVs to carry complex cargoes, including proteins, RNAs, and lipids, and to home selectively to tumor sites, positions them as versatile platforms for combinatorial therapies integrating chemotherapy, gene therapy, and immunomodulation. However, challenges such as EV heterogeneity, large-scale production, and precise targeting remain to be addressed. Advances in EV engineering, including surface modification with targeting ligands and loading of therapeutic payloads, are paving the way for clinical translation. In summary, EVs represent a multifaceted tool in prostate cancer targeted therapy, capable of delivering chemotherapeutics like docetaxel with enhanced specificity and reduced side effects, as well as mediating gene therapy through the transfer of functional miRNAs and siRNAs to regulate tumor progression and overcome resistance mechanisms. Continued research into optimizing EV-based delivery systems and understanding their interactions within the tumor microenvironment will be critical to fully realize their therapeutic potential in prostate cancer management (Zhao et al., 2021; Lavi Arab et al., 2024).

## 4 Diagnostic and therapeutic applications of extracellular vesicles in bladder cancer

### 4.1 Screening and validation of bladder cancer EV biomarkers

EVs derived from urine have emerged as a promising non-invasive source for bladder cancer (BC) biomarkers due to their direct contact with the tumor microenvironment and their cargo reflecting the molecular characteristics of originating cancer cells. Screening and validation of BC-specific EV biomarkers have focused on proteins and miRNAs that show differential expression in patients compared to controls, with potential utility in diagnosis and recurrence monitoring. For instance, specific proteins such as CD44 variant 6 (CD44v6) have been identified within urinary EVs of bladder cancer patients, serving as markers for tumor presence and progression. Lin et al. found that urinary EVs miR-516a-5p can serve as an effective biomarker for bladder cancer, with a sensitivity of 72.9 and specificity of 89.9, and an AUC of 0.79 (Teixeira-Marques et al., 2023). The specificity of these markers is enhanced by their enrichment in EVs, which protect them from degradation and provide a snapshot of the tumor molecular profile (Georgantzoglou et al., 2021; Teixeira-Marques et al., 2023).

Proteomic analyses employing mass spectrometry have been instrumental in identifying novel EV-associated proteins that are significantly upregulated in bladder cancer patients. A combined analysis of urinary EVs and tissue-exudative EVs (Te-EVs) isolated from freshly resected bladder cancer tissues revealed a substantial overlap of proteins, with 55 proteins showing increased abundance in BC patients’ urine. Among these, six proteins including heat-shock protein 90 and syndecan-1 were validated as potential urinary EV biomarkers, demonstrating significant differential expression compared to healthy controls. This dual-source approach enhances biomarker specificity by focusing on proteins directly secreted by tumor tissues (Tomiyama et al., 2021). Further, protein profiling of urinary EVs has identified alpha-2-macroglobulin (a2M) as a novel diagnostic biomarker, with elevated levels in BC patients’ urinary EVs but not in whole urine, highlighting the advantage of EV isolation to uncover tumor-specific signals otherwise masked in bulk urine samples (Lee et al., 2022). Multi-omics strategies integrating proteomics, transcriptomics, and metabolomics have delineated molecular signatures within urinary EVs that correlate with bladder cancer stage and prognosis. For example, high-throughput RNA sequencing identified a panel of long non-coding RNAs (lncRNAs) such as MALAT1 and SCARNA10 enriched in urinary EVs from BC patients, with expression levels associated with tumor grade and size. The combination of these lncRNAs with existing markers enhanced diagnostic accuracy, especially for high-grade tumors (Chen et al., 2025). Additionally, metabolic profiling using label-free optical redox ratio measurements of urinary EVs revealed altered metabolic states in bladder cancer, reflecting increased glycolytic activity in tumor-derived EVs, which may serve as a screening biomarker (Park et al., 2022). The clinical utility of EV biomarkers is further supported by studies demonstrating their prognostic value. Enumeration of tumor-derived EVs (tdEVs) in blood, alongside circulating tumor cells, stratified risk in high-risk non-muscle-invasive bladder cancer patients, correlating with time to progression and cancer-specific survival. This suggests that EV quantification could complement existing prognostic tools (Magri et al., 2024). Moreover, proteomic signatures in urinary EVs have been linked to muscle invasiveness, a key clinical parameter, with specific proteins such as HO-1 and MMP7 identified as potential markers distinguishing invasive from non-invasive disease (Steiner et al., 2025).

Despite these advances, challenges remain in standardizing EV isolation and analysis methods, which affect biomarker reproducibility and clinical translation. Recent efforts focus on optimizing sample collection, purification techniques such as ultrafiltration combined with size-exclusion chromatography, and sensitive detection assays including ELISA and targeted proteomics to improve biomarker validation (Jordaens et al., 2023; Tomiyama et al., 2022). Collectively, these studies underscore the potential of urinary EV-derived proteins and nucleic acids as robust biomarkers for bladder cancer diagnosis, staging, and monitoring, paving the way for non-invasive, accurate, and dynamic disease management.

### 4.2 EV-mediated therapeutic strategies in bladder cancer

**FIGURE 2:**
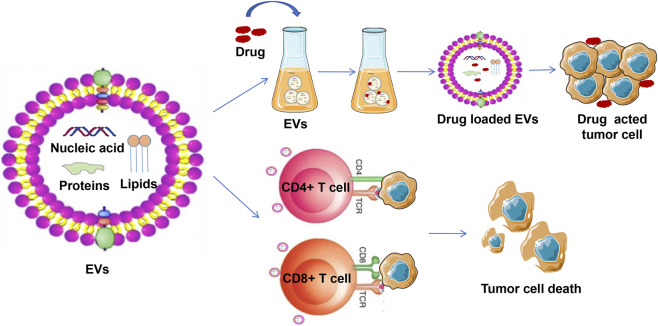
EV-mediated therapeutic strategies in bladder cancer. On the one hand, EVs are utilized as drug delivery carriers to deliver anti-tumor drugs to tumor targets, thereby enhancing therapeutic efficacy. On the other hand, EVs better eliminate tumor cells by enhancing the activity of anti-tumor cells (CD4 + T cell and CD8 + T cell).

EVs have emerged as promising tools in BC therapy due to their intrinsic ability to mediate intercellular communication by transferring diverse molecular cargos, including nucleic acids, proteins, and lipids. Two major therapeutic strategies have been explored involving EVs: their use as drug delivery vehicles to enhance chemotherapeutic efficacy and their role in modulating the tumor immune microenvironment to potentiate anti-tumor immunity (Figure 2).

#### 4.2.1 EVs as drug delivery vehicles enhancing chemotherapy accumulation and efficacy

Chemotherapy remains a cornerstone in BC treatment, yet its effectiveness is often limited by systemic toxicity and insufficient drug accumulation at tumor sites. EVs, owing to their biocompatibility, nanoscale size, and inherent targeting capabilities, have been investigated as natural drug carriers to overcome these challenges (Verma et al., 2025a). For instance, red blood cell-derived EVs (RBCEVs) have been successfully engineered to deliver IL-12 minicircle plasmids directly to MB49 bladder cancer cells, promoting localized cytokine expression that stimulates immune responses and suppresses tumor growth with minimal systemic toxicity (Wu Z. et al., 2025). Furthermore, EVs derived from chemoresistant bladder cancer cells have been shown to transfer drug resistance phenotypes to sensitive cells by modulating intracellular nucleoside metabolism and transporter proteins, highlighting the potential to target EV pathways to overcome chemoresistance (Huang C. S. et al., 2025). Notably, pharmacological modulation of EV biogenesis and release, such as inhibition of Rab27A-mediated secretion, has been proposed to influence drug resistance mechanisms. Additionally, the glycosylation status of EV surface proteins, such as sialylation, affects the expression of therapeutic targets like Nectin-4, which mediates sensitivity to enfortumab vedotin (EV), an antibody-drug conjugate used in BC therapy. Inhibition of sialylation enhances EV sensitivity and immunogenic cell death, suggesting that targeting EV surface modifications can improve drug delivery efficacy (Wu J. et al., 2025). Collectively, these findings underscore the utility of EVs as versatile nanocarriers that can enhance the accumulation and therapeutic impact of chemotherapeutic agents within bladder tumors while potentially mitigating systemic side effects.

#### 4.2.2 EVs modulating the immune microenvironment to promote anti-tumor immunity

Beyond drug delivery, EVs play a pivotal role in shaping the tumor immune microenvironment, thereby influencing the efficacy of immunotherapies. Tumor-derived EVs (TEVs) can carry major histocompatibility complex (MHC)-peptide complexes and pro-inflammatory cytokines that modulate antigen-specific CD8^+^ T-cell responses. In murine models, priming with immunogenic EVs derived from MB49 bladder cancer cells elicited robust CD8^+^ T-cell-mediated antitumor immunity, significantly delaying tumor growth (Ortiz-Bonilla et al., 2022). This immune activation was associated with increased infiltration of immune effector cells into the tumor microenvironment, suggesting that EVs can serve as natural adjuvants to enhance immune surveillance. Moreover, EVs derived from M1 macrophages engineered with molybdenum disulfide nanoparticles have demonstrated synergistic photothermal and immunotherapeutic effects, promoting M1 macrophage polarization, dendritic cell activation, and increased CD8^+^ T-cell infiltration while reducing PD-L1 expression in bladder tumors (Chuang et al., 2025). Such strategies highlight the potential of EVs to reprogram the immunosuppressive tumor milieu into an immunostimulatory state conducive to tumor eradication.

Additionally, EVs can carry microRNAs that modulate immune and tumor cell behavior. For example, bone marrow mesenchymal stem cell-derived EVs enriched with miR-139-5p suppress bladder cancer progression by targeting oncogenic pathways and activating tumor suppressor axes, thereby indirectly influencing immune responses (Xiang et al., 2022). Conversely, EVs secreted by tumor-associated M2 macrophages under carcinogen exposure deliver lncRNAs that promote metabolic reprogramming and immune evasion in bladder cancer cells, indicating the dual role of EVs in immune modulation (Zheng et al., 2025).

#### 4.2.3 Clinical implications and future directions

The integration of EV-based drug delivery and immunomodulatory strategies offers a multifaceted approach to bladder cancer therapy. EVs can be harnessed to enhance the precision and efficacy of chemotherapeutic agents, overcome drug resistance, and potentiate immune-mediated tumor clearance. Current clinical developments, such as the combination of enfortumab vedotin with immune checkpoint inhibitors, may benefit from adjunctive EV-based technologies to optimize therapeutic outcomes (Srinivasalu and Robbrecht, 2024; Bantounou et al., 2023). However, challenges remain in standardizing EV isolation, cargo loading, and targeting specificity to ensure reproducible clinical efficacy. Future research should focus on elucidating the mechanisms governing EV biogenesis and cargo selection in bladder cancer, optimizing EV engineering for targeted delivery, and conducting rigorous clinical trials to validate EV-mediated therapies. Overall, EVs represent a promising frontier in bladder cancer treatment, capable of transforming current therapeutic paradigms through enhanced drug delivery and immune modulation.

#### 4.2.4 Research progress and future directions

EVs have emerged as critical mediators in the pathogenesis and progression of various cancers, including bladder cancer, yet their precise mechanistic roles remain incompletely understood, necessitating further research to facilitate clinical translation. EVs are lipid bilayer-enclosed particles secreted by almost all cell types, including cancer cells, and carry diverse cargo such as nucleic acids, proteins, and lipids that reflect the molecular landscape of their parental cells. This cargo can modulate recipient cell behavior, influencing tumor growth, immune evasion, metastasis, and the tumor microenvironment (TME) remodeling. In bladder cancer, EVs have shown promise as non-invasive biomarkers detectable in urine, which is an accessible biofluid that reflects the state of the genitourinary tract. Recent advances have demonstrated that urinary EVs can harbor tumor-specific DNA mutations, methylation patterns, and RNA profiles that outperform traditional urine cytology in sensitivity and specificity, enabling earlier detection and better disease monitoring (Harrs et al., 2026). However, the functional contributions of EVs to bladder cancer initiation and progression are not fully delineated. For example, tumor-derived EVs can promote pre-metastatic niche formation and immune modulation by transferring oncogenic proteins and immunosuppressive molecules, but the exact signaling pathways and cargo responsible in bladder cancer require further elucidation (Shahi et al., 2021; Testa et al., 2021). Moreover, the heterogeneity of EV populations and the technical challenges in isolating and characterizing EV subtypes from urine complicate the interpretation of their biological roles and biomarker potential (Teixeira-Marques et al., 2024). Optimizing isolation protocols to improve yield and purity of EVs from urine has been shown to enhance RNA quality and biomarker detection, which is essential for clinical application (Teixeira-Marques et al., 2024). Additionally, the dynamic changes in EV cargo during bladder cancer progression and in response to therapy remain to be comprehensively characterized. The immunomodulatory roles of EVs are also increasingly recognized, with evidence suggesting that EVs carrying immune checkpoint molecules such as PD-L1 can contribute to immune evasion, highlighting potential therapeutic targets (Yamamoto et al., 2023; Chen et al., 2023). From a therapeutic perspective, engineered EVs and plant-derived EVs are being explored as novel drug delivery systems and anti-cancer agents with high biocompatibility and tumor-targeting capabilities, offering promising avenues for bladder cancer treatment (Huang Y. et al., 2025; Zhang et al., 2021). Despite these advances, translating EV research into clinical practice for bladder cancer diagnosis and therapy demands rigorous validation in large, prospective cohorts and standardization of EV isolation and analysis methods. Future directions should focus on elucidating the molecular mechanisms by which EVs contribute to bladder carcinogenesis, defining EV cargo signatures predictive of disease state and therapeutic response, and developing EV-based liquid biopsy platforms for real-time, non-invasive patient monitoring. Integration of multi-omics approaches and machine learning could enhance the sensitivity and specificity of EV biomarkers. Furthermore, investigating the interplay between EVs and the immune system may uncover novel immunotherapeutic strategies. Ultimately, a deeper understanding of EV biology in bladder cancer will accelerate the development of EV-based diagnostics and therapeutics, improving patient outcomes through precision medicine (Borea et al., 2025; Harrs et al., 2026; Nedeva and Mathivanan, 2021).

## 5 Application of extracellular vesicles in the diagnosis and treatment of renal cancer and other urogenital system tumors

### 5.1 Diagnostic value of EVs in renal cell carcinoma

EVs have emerged as promising non-invasive biomarkers for renal cell carcinoma (RCC) diagnosis, leveraging their capacity to carry tumor-specific molecular cargo such as microRNAs (miRNAs), proteins, and other nucleic acids in body fluids including blood and urine. RCC, particularly clear cell RCC (ccRCC), is a highly heterogeneous malignancy with increasing incidence and significant diagnostic challenges due to the lack of sensitive and specific biomarkers. Studies have demonstrated that EVs isolated from the blood and urine of RCC patients harbor distinct molecular signatures that reflect tumor biology and correlate with disease progression and prognosis. For instance, specific miRNAs encapsulated in serum- or urine-derived EVs, such as miR-135b-5p, miR-196b-5p, miR-200c-3p, and miR-203a-3p, have been identified as significantly upregulated in RCC patients, showing potential as early diagnostic biomarkers with reasonable accuracy in distinguishing RCC from controls, including early-stage disease (Zhang et al., 2024). Similarly, serum EV-derived miR-320d has been reported to be elevated in advanced ccRCC and is associated with recurrence and metastasis, underscoring its utility in disease monitoring (Xue et al., 2023). Beyond miRNAs, proteins such as carbonic anhydrase 9 (CA9), CD70, and CD147 have been characterized on EVs derived from ccRCC tissues and cell lines, serving as tumor-specific markers that may aid in liquid biopsy approaches (Himbert et al., 2020) Notably, CA9 expression on urinary EVs has been exploited for immunolabeling techniques to facilitate non-invasive RCC diagnosis (Mallouk et al., 2023). Moreover, bacterial DNA signatures within serum EVs, including those from Cutibacterium acnes and other bacterial taxa, have been linked to RCC presence and progression, suggesting an innovative biomarker class derived from tumor-associated microbiota (Jingushi et al., 2024; Uemura et al., 2023). The molecular cargo of EVs also reflects RCC molecular subtypes and tumor microenvironment interactions. For example, EV phosphoproteomic profiling of urinary EVs has distinguished low- and high-grade clear cell RCC, revealing upregulation of cancer-related pathways such as ErbB signaling and actin cytoskeleton regulation in aggressive tumors (Hadisurya et al., 2023). Long noncoding RNAs (lncRNAs) like AGAP2-AS1 and MALAT1 carried by EVs have been implicated in RCC progression and serve as prognostic biomarkers correlating with treatment response and survival outcomes (Fujii et al., 2025a; Jin et al., 2021). Additionally, EV-derived miRNAs such as hsa-miR-200c-3p, hsa-miR-25-3p, and hsa-miR-301a-3p have been associated with PTEN regulation and tumor aggressiveness, highlighting their dual role as diagnostic and therapeutic targets (Alves et al., 2025; Dias et al., 2020). The diagnostic potential of EVs is further enhanced by their stability in circulation and ability to reflect tumor heterogeneity more comprehensively than tissue biopsies. However, challenges remain in standardizing EV isolation and characterization methods to ensure reproducibility and clinical applicability. Integrating multi-omics analyses of EV cargo with clinical parameters and employing advanced technologies such as artificial intelligence may improve the sensitivity and specificity of EV-based diagnostics. Overall, the molecular features of EVs in blood and urine provide a rich source of RCC-specific biomarkers, including miRNAs and proteins like CA9, that not only facilitate early detection but also offer insights into tumor subtype and prognosis, paving the way for personalized management of RCC patients (Lu et al., 2023; Zhou et al., 2025; Fujii et al., 2025b).

### 5.2 Current research status of EVs in testicular cancer

Although research on EVs in testicular cancer remains relatively limited compared to other malignancies, emerging evidence highlights their significant role in tumor cell communication, metastasis, and potential clinical applications in diagnosis and therapy. Testicular germ cell tumors (TGCTs), the predominant form of testicular cancer in young men, have been the primary focus of EV-related studies. A key finding is the involvement of EVs in the intercellular transfer of microRNAs (miRNAs), particularly the miR-371-373 cluster, which is highly upregulated in TGCTs. This cluster, especially miR-371a-3p, is secreted within EVs and is being developed as a sensitive and specific biomarker for TGCT detection, with the exception of teratoma subtypes. Studies have demonstrated that TGCT-derived EVs carry these miRNAs, reflecting tumor differentiation status and intercellular communication pathways, which could be exploited for liquid biopsy-based diagnostics (Tavares et al., 2025; Mørup et al., 2020). The clinical utility of circulating EV-associated miRNAs in monitoring treatment response and disease progression is promising but requires further validation through large prospective trials.

Beyond biomarker discovery, EVs have shown therapeutic potential in mitigating testicular damage caused by chemotherapy agents such as cisplatin. Cisplatin-induced gonadotoxicity is a major concern in testicular cancer treatment, often leading to infertility. Experimental models in rats have revealed that administration of EVs can protect testicular tissue by modulating oxidative stress and autophagy pathways, preserving histoarchitecture, hormone levels, and sperm parameters. These protective effects are mediated by the antioxidant properties and regulation of autophagic activity, highlighting EVs as a novel therapeutic strategy to alleviate chemotherapy-induced reproductive toxicity (Tozak Yıldız et al., 2025). This regenerative capacity of EVs may be linked to their origin from stem cells or other testicular microenvironment components, suggesting a role in tissue repair and functional restoration. In addition, EVs participate in the crosstalk between spermatogonial stem cells (SSCs) and their supporting niche cells, such as Sertoli cells and peritubular myoid cells. Studies using porcine models have shown that EVs released by Sertoli cells are incorporated by spermatogonia, promoting their proliferation. Inhibition of exosomal release reduces SSC proliferation, indicating that EV-mediated communication is crucial for maintaining spermatogonial populations. This mechanism could be harnessed to develop feeder-free culture systems for SSC expansion, which has implications for fertility preservation in prepubertal male cancer patients who cannot bank sperm prior to gonadotoxic treatments (Thiageswaran et al., 2022). Immunologically, EVs derived from inflamed or tumor-bearing testes influence the local immune milieu. For example, EVs from testes affected by uropathogenic *Escherichia* coli-induced orchitis carry miR-155-5p, which promotes proinflammatory macrophage activation. This immunomodulatory role of EVs may also be relevant in testicular cancer, where the tumor microenvironment and immune evasion mechanisms are critical. Although direct evidence in testicular cancer is sparse, the involvement of EVs in immune regulation suggests potential avenues for immunotherapy, including the delivery of immune checkpoint inhibitors or cancer-testis antigen-targeted vaccines (Xu et al., 2023; Mehr et al., 2026). Furthermore, research in non-human models, such as giant pandas, has identified specific miRNAs (e.g., miR-331-3p) in blood and exosomal fractions as potential diagnostic markers for testicular tumors, underscoring the conserved nature of EV-associated miRNAs in testicular tumorigenesis and the translational potential of these findings (Zhu et al., 2024).

Despite these advances, the integration of artificial intelligence (AI) and multi-omics approaches in EV research for testicular cancer remains underdeveloped. Current studies are predominantly focused on miRNA biomarkers without extensive AI-driven multi-omics analysis, which limits comprehensive understanding and clinical translation. Addressing methodological challenges such as small sample sizes, batch effects, and lack of external validation will be crucial for advancing EV-based diagnostics and therapeutics in testicular cancer (Eskandar, 2025). In summary, although still in early stages, research on EVs in testicular cancer reveals their multifaceted roles in tumor biology, diagnostic biomarker development, therapeutic protection against chemotherapy toxicity, stem cell niche regulation, and immune modulation. These findings position EVs as promising candidates for improving diagnosis, treatment, and fertility preservation in testicular cancer patients, warranting further in-depth investigation and clinical validation.

### 5.3 Prospects of EVs in other urogenital tumors

EVs have shown promising potential not only in common urogenital cancers such as prostate, bladder, and renal cancers but also in rarer malignancies of the urogenital system, including penile cancer. Although research on EVs in rare urogenital tumors remains limited, their unique biological characteristics and roles in intercellular communication suggest significant opportunities for advancing both basic and clinical research in these areas. EVs carry diverse bioactive cargos-such as miRNAs, long non-coding RNAs, proteins, and lipids-that reflect the molecular landscape of their cells of origin and can modulate tumor microenvironments, immune responses, and metastatic processes. This cargo specificity provides a valuable window into the pathophysiology of rare tumors, which are often underrepresented in large-scale studies due to their low incidence. For penile cancer, a malignancy with relatively poor prognosis and limited biomarkers, EVs may offer novel diagnostic and prognostic avenues by enabling non-invasive liquid biopsies that capture tumor-derived molecules from biofluids like urine or blood. The ability of EVs to mediate hypoxia-induced changes and promote tumor progression, as observed in other cancers, further underscores their relevance in rare urogenital tumors where hypoxic microenvironments are common and contribute to aggressive phenotypes (Jiang H. et al., 2022). Moreover, mesenchymal stem cell-derived EVs have been reported to exert dual roles in genitourinary cancers by delivering therapeutic RNAs and modulating tumor microenvironments, suggesting potential applications in rare tumors for both therapy and disease monitoring (Salehpour et al., 2022). The heterogeneity of EV cargo and the influence of isolation methods on downstream analyses, as demonstrated in semen-derived EV studies, highlight the necessity for standardized protocols to ensure reproducibility and clinical applicability in rare tumor contexts (Mercadal et al., 2020). Additionally, advancements in EV isolation techniques, such as aggregation-precipitation and density-based fractionation, enable effective capture of EVs from various biofluids, facilitating biomarker discovery even in tumors with limited sample availability (Konoshenko et al., 2021; Dhondt et al., 2020). The integration of EV-based biomarkers with advanced analytical methods like deep learning and surface-enhanced Raman spectroscopy has shown superior diagnostic accuracy in common urogenital cancers, a strategy that could be extended to rare tumors to improve early detection and personalized treatment (Qian et al., 2022). Given the scarcity of clinical data on EVs in penile and other rare urogenital cancers, future research should focus on elucidating the specific EV cargo profiles associated with these tumors, understanding their roles in tumor biology, and validating their utility as diagnostic, prognostic, and therapeutic tools. This will require collaborative efforts to collect sufficient patient samples, develop sensitive detection platforms, and conduct rigorous clinical trials. Ultimately, leveraging EV biology in rare urogenital tumors holds the promise of transforming current diagnostic and therapeutic paradigms, enabling earlier diagnosis, better disease monitoring, and more effective, targeted interventions in these challenging malignancies (Verma et al., 2025b).

## 6 Technical challenges and future research priorities

EVs hold immense promise as non-invasive biomarkers and therapeutic vehicles in genitourinary cancers, yet their clinical translation is hampered by several technical challenges that demand urgent attention. A critical bottleneck lies in the optimization of EV isolation and purification methods. Currently, differential ultracentrifugation (UC) remains the most widely used technique for isolating EVs from biofluids such as urine, plasma, and serum. However, conventional UC protocols are labor-intensive, time-consuming, and often result in significant EV loss, limiting yield and reproducibility (Li J. et al., 2025). The density gradient centrifugation method is operationally labor-intensive and yields a relatively low EV recovery rate. The polymer-based precipitation method compromises purity and is significantly hindered by co-precipitation of contaminants (Li J. et al., 2025). Size exclusion chromatography exhibits limited sample throughput, high dependence on specialized instrumentation, and elevated operational costs (Li J. et al., 2025). Ultrafiltration is susceptible to membrane fouling and may induce structural damage to EVs owing to shear stress (Li J. et al., 2025). Immunoaffinity capture, while highly selective, entails substantial reagent and procedural costs and may suffer from epitope masking or antigenic heterogeneity, leading to target bias (Li J. et al., 2025). Nonetheless, these protocols still lack standardization across laboratories, contributing to variability in EV yield and purity, which complicates data comparison and clinical application. Alternative isolation methods, including microfluidic-based platforms and affinity capture techniques using cancer-specific surface markers, have shown promise in selectively enriching tumor-derived EVs with higher specificity and throughput. For example, the EVOD chip employs catalyst-free click chemistry to rapidly isolate cancer-associated EVs, enabling sensitive detection and functional analysis, and could be adapted for genitourinary malignancies (Kang et al., 2020). However, these emerging technologies require rigorous validation and scalability assessment before routine clinical use (Verma and Arora, 2025).

Equally important is the establishment of standardized detection and characterization platforms that can reliably quantify and profile EV populations in clinical samples. Multiparametric approaches such as Single Extracellular VEsicle Nanoscopy (SEVEN) combine affinity isolation with super-resolution microscopy to provide detailed phenotypic and molecular profiling of EV subpopulations, including those enriched for tumor markers like HER2. Such platforms have revealed distinct EV characteristics correlating with therapeutic resistance and disease state, underscoring their diagnostic and prognostic potential (Jiang et al., 2024). However, these high-resolution techniques are currently limited by cost, complexity, and throughput, necessitating development of more accessible and standardized assays for widespread clinical implementation. Moreover, the heterogeneity of EV cargoencompassing proteins, nucleic acids, lipids, and metabolites-requires comprehensive multi-omic characterization to identify robust and reproducible biomarkers. Pre-analytical variables such as sample collection, storage temperature, and isolation workflow significantly influence EV RNA content and integrity, as demonstrated in urinary EV studies where storage at −80 °C preserved kidney-related RNAs better than −20 °C, and different isolation methods captured distinct RNA populations (Barreiro et al., 2023). Addressing these factors through consensus guidelines will enhance biomarker reliability.

Standardization and harmonization of quality management systems for EV research and clinical application. A rigorous, well-integrated quality control framework is essential to ensure the reliability, reproducibility, and comparability of EV-related analytical results. Central to this framework is the establishment of a comprehensive reference material production, characterization, and quality assurance system. First, EV reference materials must meet stringent criteria—including analytical standardization and batch-to-batch reproducibility; physicochemical stability; biologically defined identity and function; traceable quantification; and accessibility—aligning with foundational objectives articulated in contemporary EV science (Vogel et al., 2021). Second, the EV analytical workflow spans multiple interdependent phases: biospecimen collection and handling, EV isolation, molecular and functional characterization, analytical measurement, and data interpretation. Each phase introduces potential sources of pre-analytical and analytical variability that may undermine result validity and inter-study comparability. To mitigate such variability, the International Society for Extracellular Vesicles (ISEV) and the Committee on Extracellular Vesicle Research and Application (CSEV) of the Chinese Society of Research Hospitals have jointly issued evidence-informed position statements and standardized quality control protocols. Notably, MISEV2023—the most recent ISEV guideline—provides consensus-based, stepwise recommendations for experimental design, methodological validation, and transparent data reporting, grounded in current scientific evidence and expert deliberation (Author Anonymous, 2024). Complementing this, the MIBlood-EV Quality Control (QC) Reporting Framework was developed to systematically address pre-analytical variables and implement standardized QC practices specifically for blood-derived EVs, thereby facilitating cross-laboratory benchmarking and longitudinal study reproducibility (Lucien et al., 2023). Regulatory engagement further reinforces standardization: the U.S. Food and Drug Administration (FDA) has published targeted guidance documents on liquid biopsy and EV-based biomarkers, specifying requirements for clinical trial design, analytical validation, regulatory submission, and approval pathways. In 2016, ExoDx Prostate received regulatory clearance from the U.S. FDA, becoming the first prostate cancer risk assessment tool based on EVs’s RNA (McKiernan et al., 2016).

To translate EV-based diagnostics and therapeutics into clinical practice, multicenter clinical validation studies are imperative. While promising EV biomarkers have been identified for prostate, bladder, and kidney cancers, including RNA signatures and surface proteins, most findings remain confined to single-center cohorts or preclinical models (Harrs et al., 2026). Large-scale, prospective studies with standardized protocols are needed to confirm sensitivity, specificity, and clinical utility across diverse populations. Furthermore, integration of EV analysis with other liquid biopsy components such as circulating tumor DNA and cells could improve early detection and monitoring strategies (Borea et al., 2025). From a therapeutic perspective, engineered EVs offer novel drug delivery systems with low immunogenicity and targeting capabilities, but challenges remain in scalable production, cargo loading efficiency, and safety evaluation (Zhang et al., 2021; Nedeva and Mathivanan, 2021). Future research should prioritize developing robust manufacturing processes and regulatory frameworks to facilitate clinical translation.

In summary, overcoming technical challenges in EV isolation, characterization, and standardization is essential to harness their full potential in genitourinary cancer diagnosis and therapy. Collaborative efforts to establish consensus protocols, coupled with multicenter clinical validation, will accelerate the integration of EV-based approaches into precision oncology. Continued innovation in isolation technologies and analytical platforms, alongside rigorous evaluation of pre-analytical variables, will ensure reproducibility and reliability, ultimately improving patient outcomes through earlier detection, better risk stratification, and personalized treatment strategies.

## 7 Conclusion

In summary, EVs have emerged as a transformative tool in the diagnosis and treatment of genitourinary cancers, offering distinct advantages such as non-invasiveness, high sensitivity, and specificity. From an expert perspective, the development of EV-based applications marks a significant paradigm shift in oncology, moving beyond traditional diagnostic and therapeutic modalities toward precision medicine tailored to individual patient profiles. The broad applicability of EVs across various genitourinary malignancies-including prostate, bladder, and kidney cancers-underscores their versatility, particularly in biomarker discovery and targeted drug delivery. These advances hold promise for earlier detection, improved prognostication, and more effective, less toxic treatment regimens.

However, balancing the enthusiasm for EVs with a critical appraisal of current limitations is essential. The heterogeneity in EV isolation and purification techniques remains a major impediment to reproducibility and clinical standardization. Without consensus on methodological protocols, comparing results across studies and translating findings into routine clinical practice is challenging. Furthermore, the lack of established clinical guidelines and scalable production platforms hinders the widespread adoption of EV-based diagnostics and therapeutics. Addressing these challenges requires coordinated multidisciplinary efforts integrating expertise from molecular biology, bioengineering, clinical oncology, and regulatory science.

Looking ahead, future research must prioritize elucidating the molecular mechanisms governing EV biogenesis, cargo selection, and intercellular communication within the tumor microenvironment. Such insights will refine the identification of clinically relevant biomarkers and optimize the engineering of EVs for targeted therapy. Concurrently, developing standardized, sensitive, and cost-effective detection methods is imperative to ensure reliability and facilitate regulatory approval. Large-scale, multicenter clinical trials are also crucial to validate the clinical utility of EV-based approaches and to establish evidence-based protocols for their integration into patient care. In conclusion, while extracellular vesicles represent a promising frontier in genitourinary oncology, realizing their full potential hinges on overcoming technical and translational barriers through sustained collaborative research. By harmonizing diverse research perspectives and rigorously validating EV applications, the field can advance toward precise, personalized diagnostics and therapeutics that ultimately improve patient outcomes in genitourinary cancers.
